# Engineered biomaterial strategies for controlling growth factors in tissue engineering

**DOI:** 10.1080/10717544.2020.1831104

**Published:** 2020-10-26

**Authors:** Na Guan, Zhihai Liu, Yonghui Zhao, Qiu Li, Yitao Wang

**Affiliations:** a College of Chemistry and Pharmaceutical Sciences, Qingdao Agricultural University, Qingdao, P. R. China; b Qingdao Central Hospital, The Second Affiliated Hospital of Qingdao University, Qingdao, P. R. China; c State Key Laboratory of Quality Research in Chinese Medicine, Institute of Chinese Medical Sciences, University of Macau, Macau SAR, China

**Keywords:** Growth factors, biomaterials, delivery strategies

## Abstract

Growth factors are multi-functional signaling molecules that coordinate multi-stage process of wound healing. During wound healing, growth factors are transmitted to wound environment in a positive and physiologically related way, therefore, there is a broad prospect for studying the mediated healing process through growth factors. However, growth factors (GFs) themselves have disadvantages of instability, short life, rapid inactivation of physiological conditions, low safety and easy degradation, which hinder the clinical use of GFs. Rapid development of delivery strategies for GFs has been trying to solve the instability and insecurity of GFs. Particularly, in recent years, GFs delivered by scaffolds based on biomaterials have become a hotspot in this filed. This review introduces various delivery strategies for growth factors based on new biodegradable materials, especially polysaccharides, which could provide guidance for the development of the delivery strategies for growth factors in clinic.

## 1. Introduction

Growth factors (GF) are soluble proteins, which can participate in cell growth, proliferation, differentiation, migration, etc (Qian et al., 2017; Wang et al., 2017). They are derived from kinds of cells, including macrophages, fibroblasts, and so on, which coordinate immune responses, angiogenesis, and wound healing (Gallego-Muñoz et al., 2017; Duncan et al., 2018; Ishihara et al., 2018). They exert functions via specifically binding with receptors on the surface of cell membrane and participate in cell proliferation, differentiation and migration (Surmacz, 2003; Davydova et al., 2016; Cipitria & Salmeron-Sanchez, 2017). They have important applications in wound healing, skin tissue engineering, cartilage tissue engineering, and bone tissue engineering (Jia et al., 2016; Millette et al., 2006; Caballero Aguilar et al., 2019). For instance, vascular endothelial growth factor (VEGF) and fibroblast growth factor (FGF) families can promote cell migration and proliferation of endothelial cells; platelet-derived growth factor-BB (PDGF-BB) stimulate pericyte adhesion and blood vessel maturation, including stem cell recruitment and granulation tissue formation (Hellberg et al., 2010); The hepatocyte growth factor (HGF) can modulate the proliferation, migration and differentiation of mesenchymal stem cells (Catizone et al., 2006). Growth factors have various specific biological characteristics so that clinical applications are also different (Table 1).

**Table 1.** Growth factors and their clinical applications in clinical practice.

Growth factors	Clinical application
Vascular endothelial growth factor (VEGF)	Regeneration of epithelial tissue repair, treatment of peptic ulcer (Niu et al., 2019)
Fibroblast growth factor (FGF)	Bone repair, angiogenesis, nerve growth, treatment of chronic complications of diabetes (Figueroa et al., 2019)
Hepatocyte growth factor (HGF)	Chronic liver injury repair, stimulate angiogenesis HGF participate in angiogenesis, morphology, organogenesis, bone restructuring, nerve induction (Vanderwerff et al., 2019)
Insulin-like growth factor (IGF)	cell differentiation, proliferation, individual growth and development (Mancarella et al., 2016)
Transforming growth factor (TGF)	the growth, differentiation, apoptosis and immune regulation of many kinds of cells (Onodera et al., 2019)
Nerve growth factor (NGF)	The treatment effect of neurodegenerative disease is especially remarkable, which is also used to speed burn recovery and reduce the side effects of chemotherapy and radiation (Aloe et al., 2016)

Growth factors facilitate the proliferation and differentiation of progenitor and stem cells, as well as directly induce growth of differentiated cells such as hepatocytes in the liver or osteoblasts in bone (Uebersax et al., 2009). They also have an important regulatory role in human immunity, hematopoietic regulation, tumorigenesis, inflammatory infection, wound healing, angiogenesis, cell differentiation, cell apoptosis, morphogenesis, and embryo formation (Arisaka & Yui, 2019; Chu et al., 2019; Evans et al., 2019). Growth factors mainly include epidermal growth factor (EGF), platelet-derived growth factor (PDGF), fibroblast growth factor (FGF), and transforming growth factor beta (TGF-β) families (Goh et al., 2016). In the past decades, many researches try to uncover more GFs functions in biomedical science and delivery of GFs using biomaterials has become a pretty hot topic. Currently, numerous researches have shown that scaffolds based on biomaterials could delivery growth factors to promote tissue repair and regeneration at a faster rate (Venkatesan et al., 2017). However, growth factors for clinical applications achieved little clinical success, which were still limited by the instability and safety of GFs (Nicoletti et al., 2019). The key to overcoming the challenges lies in better deliver GFs, maintain their activities and alleviate their adverse effects.

**Figure 1.**
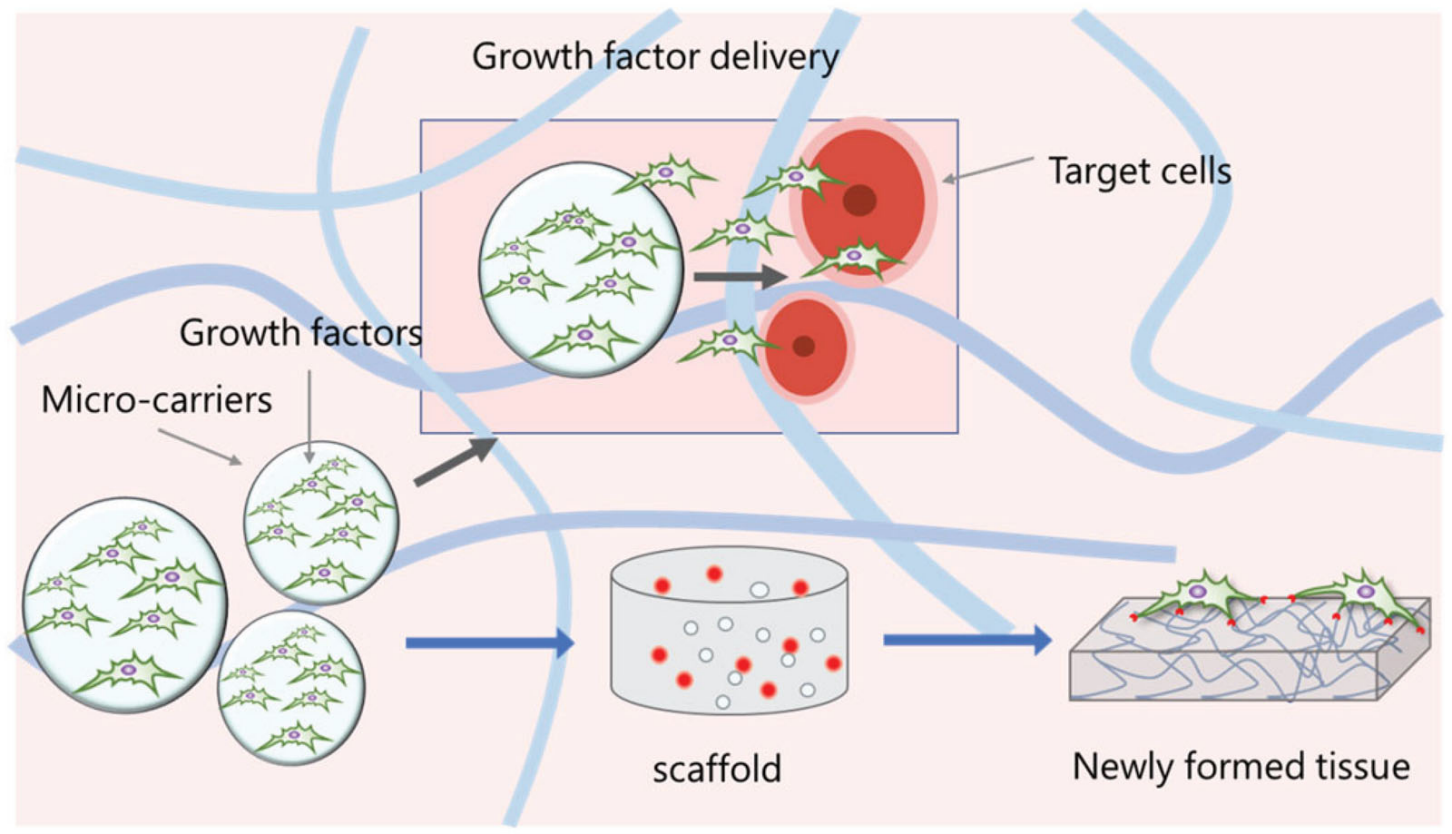
Growth factor delivery in tissue engineering (Aguilar et al., 2019).

Although there are extensive studies on the safety and efficacy of growth factors in clinical trials, there are significant limitations associated with the use of growth factors, including ectopic bone formation, osteolysis, pain and swelling. Given the long-term regeneration process in live organism, it is difficult to resolve the contradiction between efficacy and adverse reactions in current studies. Clinically, excessive soluble protein or even milligram level (one million times the concentration required for physiological regeneration) is usually used to ensure clinical effectiveness (Caballero Aguilar et al., 2019). However, high doses of soluble growth factors will inevitably spread to nearby tissues or blood circulation, bringing many adverse effects (Caballero Aguilar et al., 2019). Although many carriers have adapted to control initial burst and rapid clearance of GFs, safety and efficacy of GFs remain mutually exclusive. The cost-effectiveness and legal issues associated with soluble recombinant growth factors are now challenging in the field of medicine. Precise delivery and safe release of growth factors have emerged as a promising spot in tissue engineering. Biomacromolecules including various polysaccharides have been widely studied as novel biomaterial scaffolds for GF delivery in regenerative medicine (Shelke et al., 2014; Degirolamo et al., 2016; Zhang et al., 2018). To date, researchers have designed a variety of biomaterial-based delivery systems to enhance tissue regeneration by maintaining high levels of GFs over a long period of time *in situ* (Hendrikse et al., 2018; Zheng et al., 2018; Guo et al., 2019). The polysaccharides and other biomacromolecules were developed into various nanofibers, scaffolds, sponges, microspheres, nanoparticles, and composites, which play a crucial role in the delivery of growth factors (Kumar et al., 2018; Miao et al., 2018; Tiwari et al., 2018). On the one hand, because of their biochemical similarity with human extracellular matrix (ECM) components, these biomaterials are readily recognized and tolerated by native body, on the other hand, natural biomaterials inherit numerous advantages including natural abundance and adequate capacity for chemical modification to meet varying technological requirements. In this review, we will focus on the biomaterials-based approaches to improve the delivery of GFs in tissue engineering, with the emphasis on the control of GFs activities using natural polysaccharides (Figure 1).

## 2. Applications of natural polysaccharides in tissue engineering

Natural polysaccharides for tissue engineering are widely used for treating human damaged organs and dying tissues, discovering tissue engineered substitutes is the key in clinic (Pearman et al., 2019; Vandghanooni & Eskandani, 2019). However, there are still huge challenges in applications, the scaffolds must provide appropriate mechanical and chemical properties to guide appropriate cell behavior. Further, they enable regeneration and replacement of the necessary biological functions. The extracellular matrix of the implanted area is the ideal template and ECM has many unique properties including guiding cell migration, adhesion and differentiation, at the same time, it can be degraded and rebuilt as needed. Natural polysaccharides exhibit structure and function similarity with ECM (Hinz, 2015; Erdogan & Webb, 2017), as the extracellular matrix mainly consists of protein polysaccharides, glycosaccharides, glycoproteins, and glycolipids. Meanwhile, natural polysaccharides have good biocompatibility and biodegradability, which are more and more widely used in the field of tissue engineering (Le Dour et al., 2017; Yan et al., 2018). The natural polysaccharide, like cellulose, has the strong mechanical strength and biocompatibility, which can be applied for tissue engineering, including engineering vascular tissue, bone, cartilage, skeletal muscle, and cardiac muscle. Additionally, cellulose also can be used to establish nano-fibrous carrier for liver cells and create tubes for regeneration. The chitosan as a natural polysaccharide also can be modified into scaffolds in tissue engineering, which exhibits the significant effect on supporting and aiding the generation of extracellular matrix *in vivo*. The alginate derived from sea can be changed into gels and applied for cell transplantation in tissue engineering. Therefore, a large volume of research suggests that polysaccharides may interact with tissue cells and regulate these cells in diverse ways. Then the polysaccharides can be modified into versatile scaffolds in tissue engineering (Li et al., 2016; Wang et al., 2017) (Table 2).

**Table 2.** Various polysaccharides from nature.

Polysaccharides	Source	Functions
Starch	Plants	Storage, drug adjuvant
Chitosan and chitin	Animals	Tissue scaffolds
Pectin	Plants	Food additives
Hyaluronic acid	Animals	Animal tissue structure, therapeutic agents
Cellulose	plants	Cell structure, food additives
Heparin and heparin sulfate	Animals	Animals tissue structure, therapeutic
Alginate	Microorganism	Drug adjuvant
Carrageenan	Microorganism	Food additives

### 2.1. Animal-derived polysaccharides for controlling growth factors

Polysaccharides extracted from animals play a vital role in growth factors delivery. The extracellular matrix in animal tissues is composed of interlocking reticular structures, which is filled with heteropolysaccharides, fibrins, and gelatinous substances, supporting cell adhesion, and providing porous pathways for the diffusion of nutrients and oxygen for individual cells (Bhaskar et al., 2012). For instance, heteropolysaccharides, called glycosaminoglycans, are repeated by disaccharide units, which include hyaluronic acid, heparin, heparin sulfate, chondroitin sulfate (CS), piperin sulfate, and keratin sulfate (DeAngelis, 2012). Additionally, chitosan and chitin derived from animals are also widely used for growth factors delivery in tissue engineering.

#### 2.1.1. Chitosan and chitin

**Figure 2.**
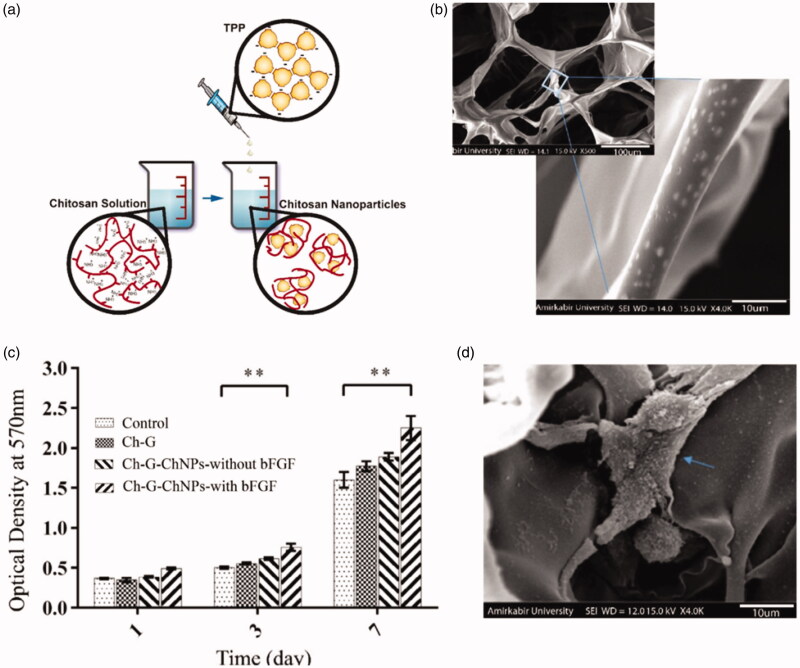
(a) A schematic of chitosan nanoparticles prepared by ionic gelation method; (b) SEM showed the chitosan nanoparticles were incorporated within the chitosan-gelatin scaffold; (c) MTT assay for fibroblast cells cultured on scaffolds after 1, 3 and 7 days (**p < .01); (d) SEM image of fibroblast cells attached to the chitosan nanoparticles loading growth factors in chitosan-gelatin scaffold on day 7 after culture; The blue arrow stands for the attached cells (Azizian et al., 2018).

Chitosan is a deacetylated derivative of chitin extracted from arthropods, usually in the form of particles, flakes, or powders. Both chitosan and chitin are linear polysaccharides consisting of repeated n-acetyl-2-amino-2-deoxyd-glucose (n-acetyl) and 2-amino-2-deoxyd-d-glucose residues (n-deacetyl, amino) (Venkatesan et al., 2015). Numerous studies have shown that chitosan and chitin can be used in a variety of applications in tissue engineering (Yilgor et al., 2009; Liu et al., 2016; Pangon et al., 2016; Islam et al., 2017; Ahsan et al., 2018). They can be designed into a variety of structures such as gels, membranes nanofibers, nanoparticle and sponges (Singh et al., 2017; Smirnova et al., 2019; Zubillaga et al., 2020). In combination with properties such as its good biocompatibility, biodegradability, nontoxic, anti-inflammatory, adhesion, antibacterial and nerve protection, chitosan can be made into a suitable candidate for growth factors delivery (Rajam et al., 2011). Furthermore, being one of the few natural polycationic polysaccharides, it permits chitosan to have many electrostatic interactions that can be leveraged for the production of biomaterials (such as in layer-by-layer polyelectrolyte assemblies). These chitosan based materials can easily be made into the shape needed for tissue support and regeneration scaffolds. For example, Azizian et al. claimed that chitosan nanoparticles loaded with basic fibroblast growth factor (bFGF) and bovine serum albumin (BSA) was introduced into the chitosan-gelatin scaffold, the experimental results showed that chitosan nanoparticles significantly affected the property of the scaffold, which could continuously release growth factors to promote the proliferation of fibroblasts (Azizian et al., 2018). This experiment incited a growing interest for chitosan - gelatin scaffold and provided a reference for delivering growth factors, which could be widely used in bone tissue engineering and provide a good matrix for bone tissue regeneration and repair. Venkatesan et al. reported that chitosan had the ability to increase alkaline phosphatase activity, enhance serum calcium concentration, and promote osteoblast differentiation and bmp-2 expression in rabbit models (Venkatesan et al., 2017). And Gohil et al. demonstrated that chitosan hydrophobic gel has great potential in delivering growth factors and proteins. The presence of amino and hydroxyl functional groups in chitosan plays an important role in the release of growth factors in the extracellular matrix. Chitosan combines with negatively charged biomolecules through electrostatic action of positive charge generated by amino protonation in the chain to control the release of encapsulating proteins (Gohil et al., 2017). Additionally, Song et al. (Oh & Lee, 2019) indicated that the combination of chitosan and silica dressing loaded with keratinocyte growth factor (KGF) had the characteristics of continuous KGF release. The results have shown that the dressing promotes skin regeneration. The chitosan scaffold found in the present study is mainly used to load growth factors, which can also be used in combination with other materials. The combination of heparin and chitosan to form scaffold - supporting nerve growth factor (NGF) was used to promote nerve regeneration. Li et al. (2017) found that heparin/chitosan scaffold loaded with NGF significantly improved cell adhesion and proliferation. More importantly, the NGF heparin/chitosan scaffold can effectively promote cell morphological development. Sara et al. (Azizian et al., 2018) prepared the chitosan nanoparticles, which were synthesized by ionic gelation method (average size 266 nm), loaded with BSA-bFGF, and were incorporated into the 3 D porous chitosan-gelatin scaffold. The system achieved the sustained release of growth factors from the scaffold and extremely promoted fibroblast cell proliferation *in vitro* (Figure 2).

#### 2.1.2. Hyaluronic acid

Hyaluronic acid (HA), an acidic polysaccharide, was isolated from bovine vitreous in 1934 by Meyer et al., a professor of ophthalmology at Columbia University, which is a non-sulfated glycosaminoglycan and major component of the extracellular matrix (Necas et al., 2008; Mangla et al., 2017). Hyaluronan (or hyaluronic acid) is a linear poly-saccharide made up of alternating β-(1 → 4)-D-glucuronic acid and β-(1 → 3)-N-acetyl-D-glucosamine residues that is found in cartilage, synovial fluid, and skin (Tchobanian et al., 2019). It is a kind of natural hydrophilic anion polymer, which can regulate the concentration of positive and negative ions around. Hyaluronan presents itself as a promising biomaterial in medical and cosmetic fields (Highley et al., 2016; Li et al., 2016;; Wickens et al., 2017). Furthermore, hyaluronic acid has multiple hydroxyl and amino groups on its surface, which can sequester growth factors and chemically modified by block polymers. More importantly, HA both has natural biocompatibility and biodegradability, which is also soluble in water, as well as showing fast absorption, short time of retention in tissues, fast degradation (Benedetti et al., 1993; Tan et al., 2009). In sum, hyaluronic acid has a promising prospect in the future for growth factors delivery.

**Figure 3.**
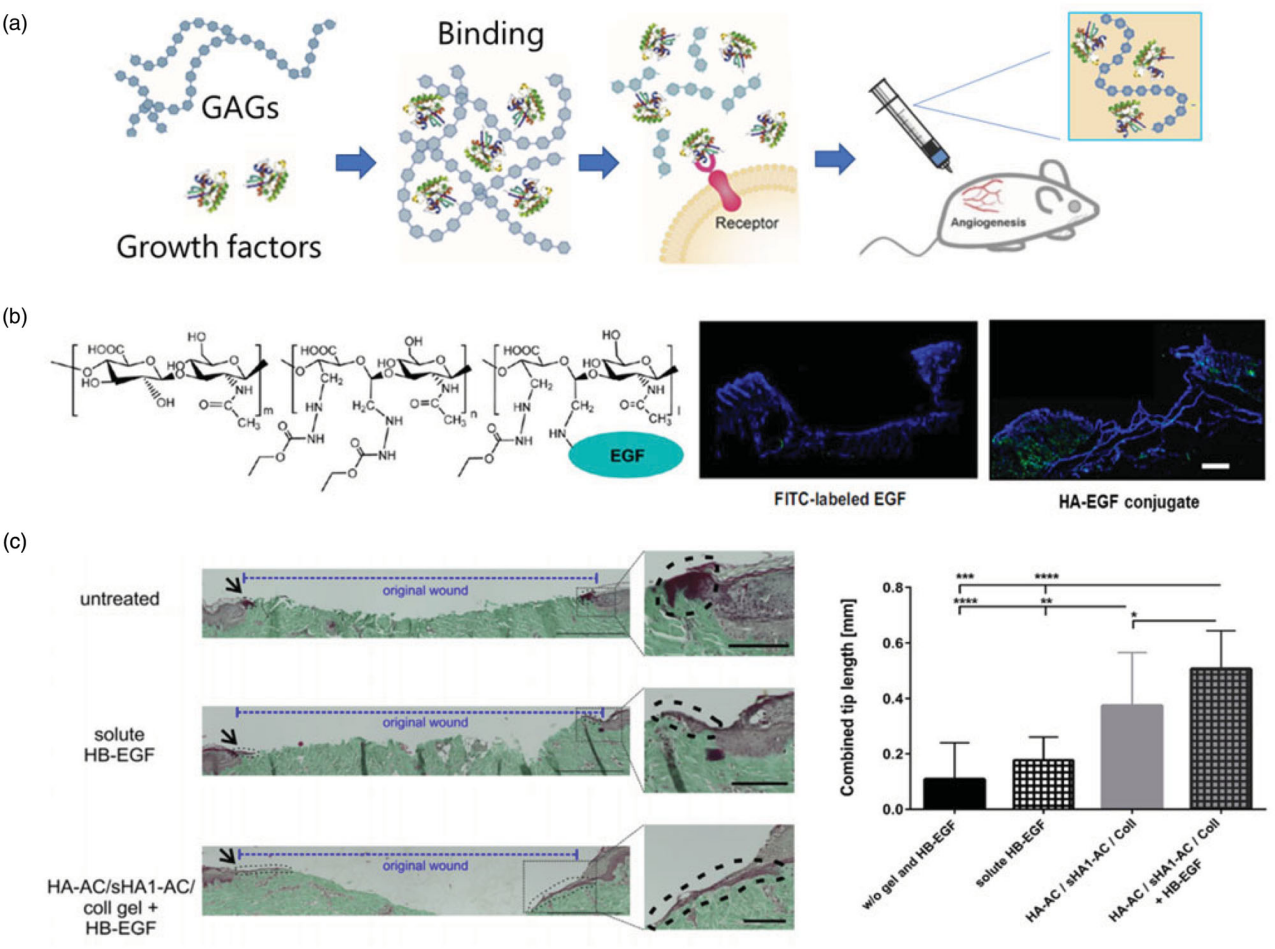
(a) Growth factors binding with GAGs (Hachim et al., 2019). (b) the chemical structure of hyaluronate-epidermal growth factor (HA-EGF) conjugates; the fluorescence graphs of the degradation behavior of EGF and HA-EGF conjugate in HA films after topical application of FITC-labelled samples on wounded tissues (Niu et al., 2019). (c) HB-EGF released from sHA1-AC-containing hydrogels promotes epidermal tip formation and the results were measured by Masson-Goldner Trichrome staining. Bars: 500 μm; 100 μm (insets). The combined tip lengths of both wound sites were shown (*n* = 3 with 3–4 sections per condition *p < .05, **p < .01, ***p < .005, ****p < .001) (Thönes et al., 2019).

Hyaluronic acid is an important component of extracellular matrix of vertebrates (Mercer et al., 2013). It has been reported that hyaluronic acid owning high molecular weight inhibits angiogenesis, while hyaluronic acid at low molecular weight stimulates endothelial cell proliferation and migration (Thönes et al., 2019). Because hyaluronic acid lacks sulfonic groups, it has a relative weak affinity for growth factors. The affinity of hyaluronic acid to growth factors can be improved by chemical modification, and the function of stabilizing growth factors and continuously controllable release can be realized. The modified HA achieves the functions of stabilizing growth factors and controllable release. For example, Zhou et al. (2016) adopted a simple method for preparing silk fibroin (SF) and hyaluronic acid (HA) composite film. The composite film loaded with VEGF (vascular endothelial growth factor), and the experimental results showed that the modified composite membrane can accelerate the release of VEGF, exhibiting a huge potential for growth factor delivery. In addition, the results showed that lowering the temperature and adding HA led to the rapid release of VEGF (vascular endothelial growth factor) in the HA/SF membrane. Su et al. (2014) developed a scaffold in the hope of replacing skin. The results showed that HA increased the adsorption of EGF and maintained the release of EGF from the scaffolds. Scanning tunneling microscope (STM) showed stenting was stable. Animal studies showed that mice treated with scaffolds containing EGF and HA healed well, and wounds treated with scaffolds containing both HA and EGF formed skin attachment 20 days after surgery. Thones et al. (2019) indicated that sulfated HA in hydrogel loaded with HB-EGF growth factor could induce the continuous release of HB-EGF for wound healing. HB-EGF is a growth factor that supports wound repair by activating epidermal keratinocytes and dermal fibroblasts. The experimental results showed that hyaluronic acid/collagen hydrogel containing sulfureted hyaluronic acid could significantly improve the efficacy of HB-EGF hydrogel. Functionalized hydrogel containing sulfur HA was a scaffold that effectively released growth factors (Figure 3).

#### 2.1.3. Heparin and heparin sulfate

**Figure 4.**
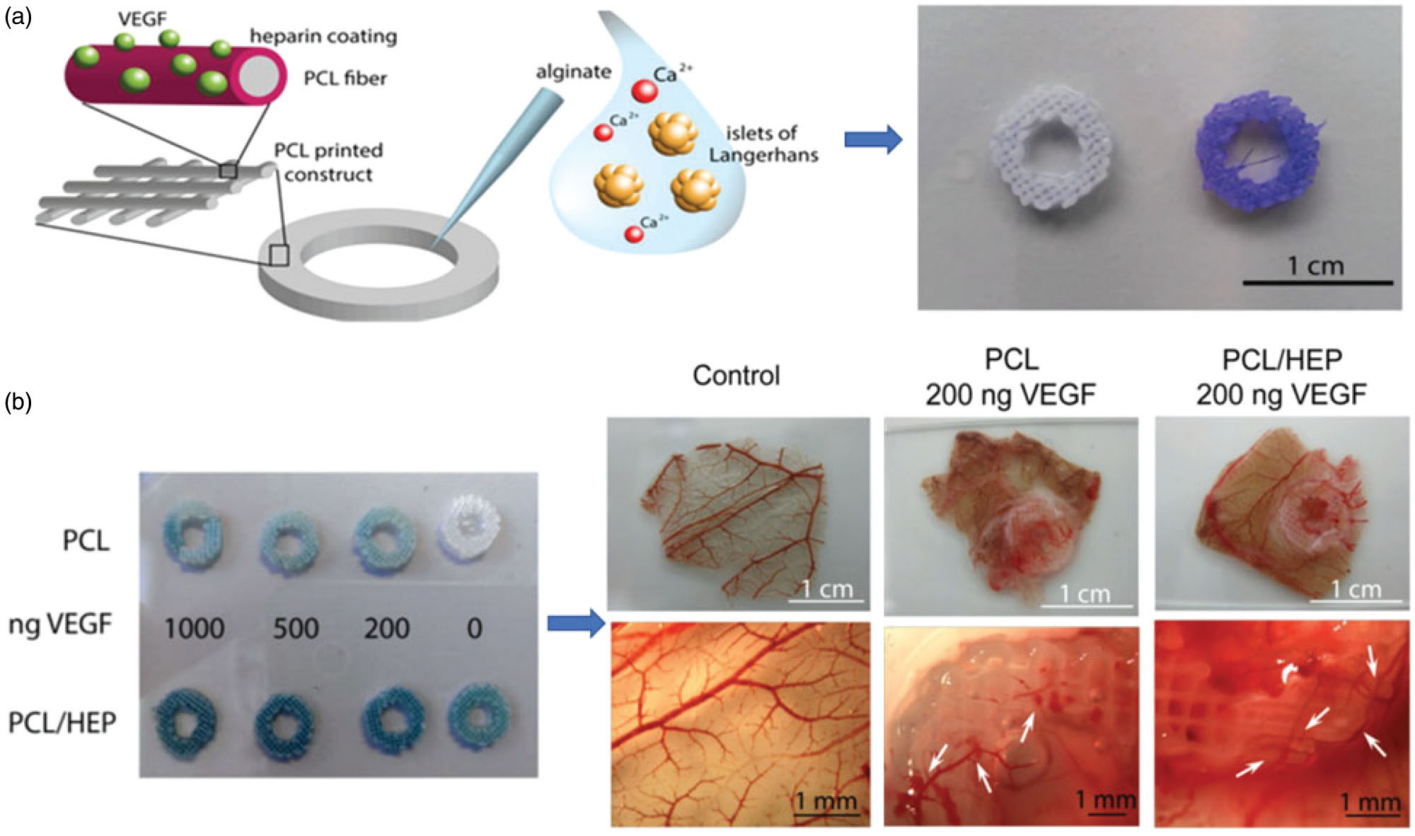
(a) A schematic of showing the functionalized surface of PCL constructs binding VEGF. And the PCL and PCL/heparin plotted scaffolds stained with Azure II. (b) Graphs of neovascularization in PCL-VEGF and PCL-heparin-VEGF (Marchioli et al., 2016).

Glycosaminoglycans (GAGs) represent a very promising group of components, which they could be functionally engineered and are well tolerated by the recipient tissues due to their relative immunological inertness (Dao et al., 2018). Both heparin and heparin sulfate are glycosaminoglycans (GAGs), which consist of repeated units of sulfonated hexatonic acid (1 → 4)-*D*-glucosamine (Hettiaratchi et al., 2017; Mohammadi et al., 2019). Heparin can be synthesized and stored in mast cells, while heparin sulfate, as a proteoglycan, is mainly present in the extracellular matrix of cells and tissues. Interestingly, heparin combines with many growth factors, which possesses a heparin-binding domain. This affinity-based binding ability slows down the diffusion of immobilized growth factors thus tempering the initial burst (Brasil et al., 2016). The heparin-based delivery systems can also prevent growth factors from enzymatic degradation thereby potentiating their biological functions (Choi et al., 2016). Heparin has a high affinity with various growth factors, which can regulate bioavailability, signal transduction and stability of growth factors. Heparin has the highest electronegative charge, which mediates its important biological roles as a multivalent binding agent for many proteins, including antithrombin III (to mediate thrombosis) and growth factors (to protect against degradation and potentiate receptor binding) (Nie et al., 2007). All these studies using covalently bound heparin were designed to enhance the biological properties of the hydrogel. Heparin-based delivery systems can also prevent enzymatic degradation of binding growth factors so that enhancing their biological functions. For example, levinson et al. (Caballero Aguilar et al., 2019) has previously described a system in which heparin was combined with hydrogel made by hyaluronic acid (HA) and transglutaminase (TG), the system showed the ability of controllable release of TGF-β (transforming growth factor). Another research developed a modified heparin impregnated with a light crosslinking alginate brine gel. After modified with heparin, the biodegradation properties of gel, swelling ratio and the modulus of elasticity were almost unchanged (Jeon et al., 2011). This controllable growth factor delivery system with independently controllable physical and cell adhesion properties provides a powerful therapeutic approach for a variety of therapeutic applications. Choi et al. (2016) designed a system in which novel metal scaffold is fabricated by immobilizing heparin on the HA coated Co-Cr surface and loaded with vascular endothelial growth factor (VEGF) as well as hepatocyte growth factor (HGF). The results demonstrated that scaffolds modified with HA and heparin showed the capacity of rapid recovery of endothelial cells. Tang et al. (2010) presented a system in which multi-functional delivery of basic fibroblast growth factor (bFGF) was incorporated with heparin-functionalized chitosan (CS)/poly(g-glutamic acid) (g-PGA) nanoparticles (HP-CS/g-PGA nanoparticles). With the increase of heparin content, the average particle size and bFGF loading efficiency are increasing. This delivery system may be a potential strategy for regeneration. Giulia et al. developed a novel 3 D scaffold platform consisting of a 3 D ring-shaped polycaprolactone (PCL) scaffold with heparinized surface to electrostatically bind vascular endothelial growth factor (VEGF) that can significantly promote vascularization. The scaffold demonstrated that heparin immobilization could be used for controlled delivery of VEGF and avoiding degradation (Marchioli et al., 2016) (Figure 4).

### 2.2. Microbial polysaccharide for controlling growth factors

Polysaccharides obtained from the microorganism are also one kind of the major polysaccharides existed in nature. Some of Microbial polysaccharides including the glycogen act as storage compound. Moreover, microbial polysaccharides show a potential prospect in medical, pharmaceutical and biomedical fields, which can be used as wound dressings, biomaterials, and tissue scaffolds.

#### 2.2.1. Alginate

Alginate is a natural polysaccharide extracted from brown algae and is composed of various proportions of β-d-mannuronic acid (M) and α-l-guluronic acid (G) residues (Gu et al., 2004). Common alginate usually originates from algae and has a high degree of physicochemical heterogeneity, which can affect its quality and induce different applications (Mhanna et al., 2017). Alginate has many excellent properties, such as biocompatibility, low toxicity, low cost and has been widely studied in the field of biomedicine. Alginate gel induced by bivalent cation can be used for wound healing, therapeutic drugs, protein delivery and cell transplantation (Li et al., 2018). The main advantage of alginate is that stimulate the extracellular matrix and create a moist environment so that reduces the risk of bacterial infection in wounds and accelerates wound healing. What’s more, alginate hydrogel has also been used in cell transplantation in tissue engineering (Reakasame & Boccaccini, 2018). It transports cells to specific sites and provides an artificial matrix for new blood vessels. Alginate gels can also be administrated orally or injected into the body and can also be used in pharmaceutical fields (Kalaf et al., 2016; Yan et al., 2016).

**Figure 5.**
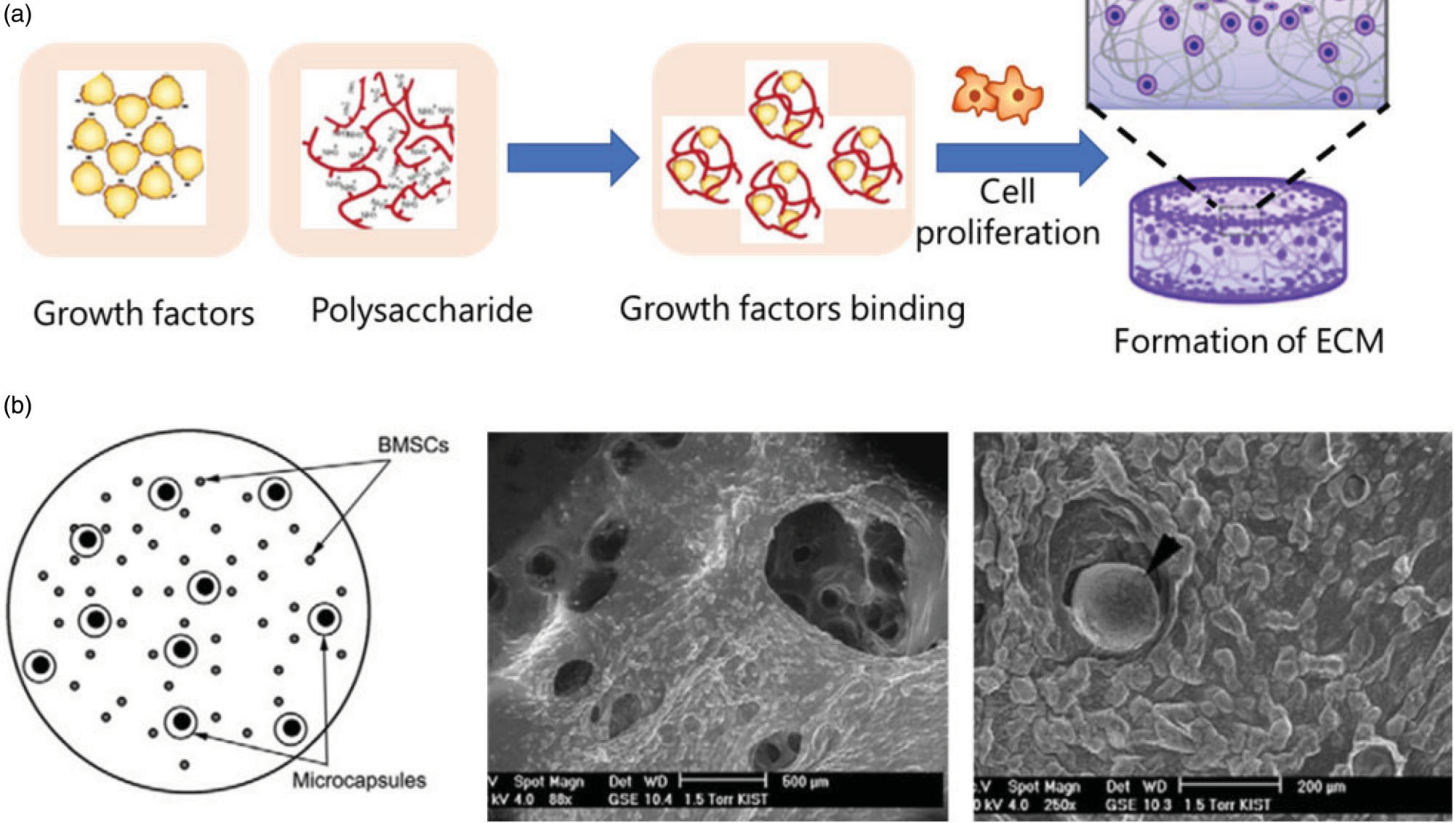
(a) Growth factors binding with polysaccharides for cell proliferation; (b) A schematic of encapsulation of rat BMSCs in the alginate hydrogel, along with BMP-2/Dex-loaded microcapsules; Then the surface morphology of alginate scaffolds was presented using SEM and a microcapsule was exposed on the surface of the alginate construct (Choi et al., 2010).

Vascular endothelial growth factor (VEGF) is a potent pro-angiogenic signal transduction molecule (Jha et al., 2017; Roskoski, 2017). At an appropriate dosage, VEGF can up-regulate angiogenesis by signaling endothelial cells to undergo proliferation, migration and differentiation into new blood vessels (Gu et al., 2004). For example, Choi et al. (2010) designed a system in which microcapsules made by PLGA and alginate were used for double-layer growth factor delivery, the bioactive growth factors were encapsulated in microcapsules. The experimental results showed that the system controlled the release of growth factors. Another example from Gu et al. (2004) claimed that VEGF was encapsulated in calcium alginate beads using the extrusion/external gelation method, and was subsequently released in PBS and in serum media. The results showed that they obtained controlled release of VEGF *in vitro*. Additionally, alginate also could be modified with different degrees of sulfation using SO_3_/pyridine and obtained the ability of binding with FGF-2. The degree of modification mattered, and polysaccharides with higher sulfation degrees exhibited a stronger affinity for FGF-2 (Mhanna et al., 2017) (Figure 5).

#### 2.2.2. Dextran

Dextran is a high molecular-weight polysaccharide, consisted of α-1, 6 linking glucose of the backbone, α-1, 4 linking glucose of side chain. It can be extracted from different microbial strain owning different structures. It can be cross-linked for the separation and purification of proteins. Dextran possessing well biocompatibility can also be applied as the plasma expander in clinic. As for delivering growth factors, Zhang et al. (2019) established a dextran/poly(lactic-co-glycolic acid)-combined microsphere system for sustained delivery of vascular endothelial growth factor us for therapeutic neovascularization. In this study, the VEGF-loaded dextran microparticles were prepared and then they were encapsulated into poly(lactic-co-glycolic acid) (PLGA) microspheres in order to obtain VEGF–dextran–PLGA microspheres. Finally, a novel growth factor sustained-release system based on the combination of protein-loaded dextran microparticles and PLGA microspheres was fabricated, and achieved a great progress in promoting mature vessel formation in a rat hind-limb ischemic model.

### 2.3. Botanical polysaccharides for controlling growth factors

Botanical polysaccharides comply with many requirements of pharmaceutical excipients such as nontoxicity, stability, availability and renewability, they are extensively investigated for use in solid oral dosage forms (Wang et al., 2017; Chen et al., 2018). Meanwhile, botanical polysaccharides have been used for industrial applications, e.g. pharmaceuticals, biomaterials, food stuff, nutrition, and biofuels (Jiao et al., 2016; Yin et al., 2019). Furthermore, polysaccharides with varying physicochemical properties can be extracted from plants at a relatively low cost and can be chemically modified to suit specific requirements (Beneke et al., 2009). Numerous researches indicate that polysaccharides can be used in biomedical science and have a diverse of therapeutic properties, such as antioxidant activity (Chen et al., 2019), immune activity (Miandare et al., 2017). It has been proved by experiments that adding active polysaccharides in feed can significantly improve the immune function of livestock and poultry. It can not only promote the development of immune organs, activate lymphocytes, but also promote the generation of antibodies and reduce animal mortality (Debele et al., 2016). Botanical polysaccharides have comprehensive physiological activity in human body and much studied indicated that polysaccharides can potently regulate the immune system. The main immune cell- macrophages can be activated by many botanical polysaccharides through specific membrane receptors. Based on this property, botanical polysaccharides have a promising prospect in tissue engineering. For instance, the *Bletilla striata* polysaccharide is obtained from *B. striata*, which composed of α-mannose, β-mannose and β-glucose at the mole ratio of 2.4:1. It can interact with macrophages and widely used in tissue regeneration. It was found that after the wound treated with BSP gel based on this polysaccharide, it was proved to control the inflammatory responses and accelerate the wound closure. Therefore, botanical polysaccharides are promising therapeutic natural materials, which can be widely used in biomedical science. But the studies of botanical polysaccharides on controlling growth factors are relative less and we summarize here.

#### 2.3.1. Cellulose

Cellulose is the most abundant renewable polymer in nature, which is isolated from the secondary cell wall of plant cells, accounting for 20% ∼ 30% of the dry weight of plant cells (Rao et al., 2019). It plays a vital role in the field of functional foods. For instance, a variety of indigestible plant polysaccharides including cellulose, hemicelluloses, pectins, oligosaccharides, gums, were defined as the dietary fiber by the Food and Agriculture Organization (FAO). Among these, cellulose and hemicellulose can directly stimulate the bowel movement, which is the most widely spreading polymeric material in nature, is a fibrous, tough, water-insoluble material (Lynam et al., 2017; Xu et al., 2018). The basic unit of cellulose macromolecules is the glycan consisting of β-1, 4-glycoside bonds (Wang et al., 2016, 2018). Cellulose semi-synthetic derivatives are widely used in cosmetic and pharmaceutical fields, as well as used as dialysis membrane and biosensor (Amsden, 2015).

As for controlling growth factors, Roger et al. synthesized a delivery vehicle composed of an injectable hydrogel of hyaluronan (HA) and methyl cellulose (MC). Methyl cellulose was modified using thiol-maleimide and biotin–streptavidin chemistry to covalently bind the cell adhesive peptide, glycine–arginine–glycine–aspartic acid–serine (GRGDS), and the oligodendrocyte-differentiating factor, recombinant platelet-derived growth factor A (rPDGF-A). The experimental results indicated that this scaffold could induce more NSPCs differentiating into oligodendrocytes, compared with control groups (Tam et al., 2012).

#### 2.3.2. Polysaccharides from Chinese medicinal herbs—new prospects for controlling growth factors

Of these fractions in herbal medicines, polysaccharides have been identified as major active ingredients, responsible for various pharmacological activities. Although the detailed mechanism of these effects is under exploration, the immunostimulatory activities of many polysaccharides are confirmed. Botanical polysaccharides can interact with macrophage receptors on the surface and activate different signaling pathways. For example, it can enhance the phagocytic activity of macrophages and promote the secretion of cytokines (Pearman et al., 2019). In the past decades, many polysaccharides were obtained from Chinese medicinal herbs; they have received massive attention as promising biomaterials in tissue engineering because of their biocompatible, safe and biodegradable properties.

**Figure 6.**
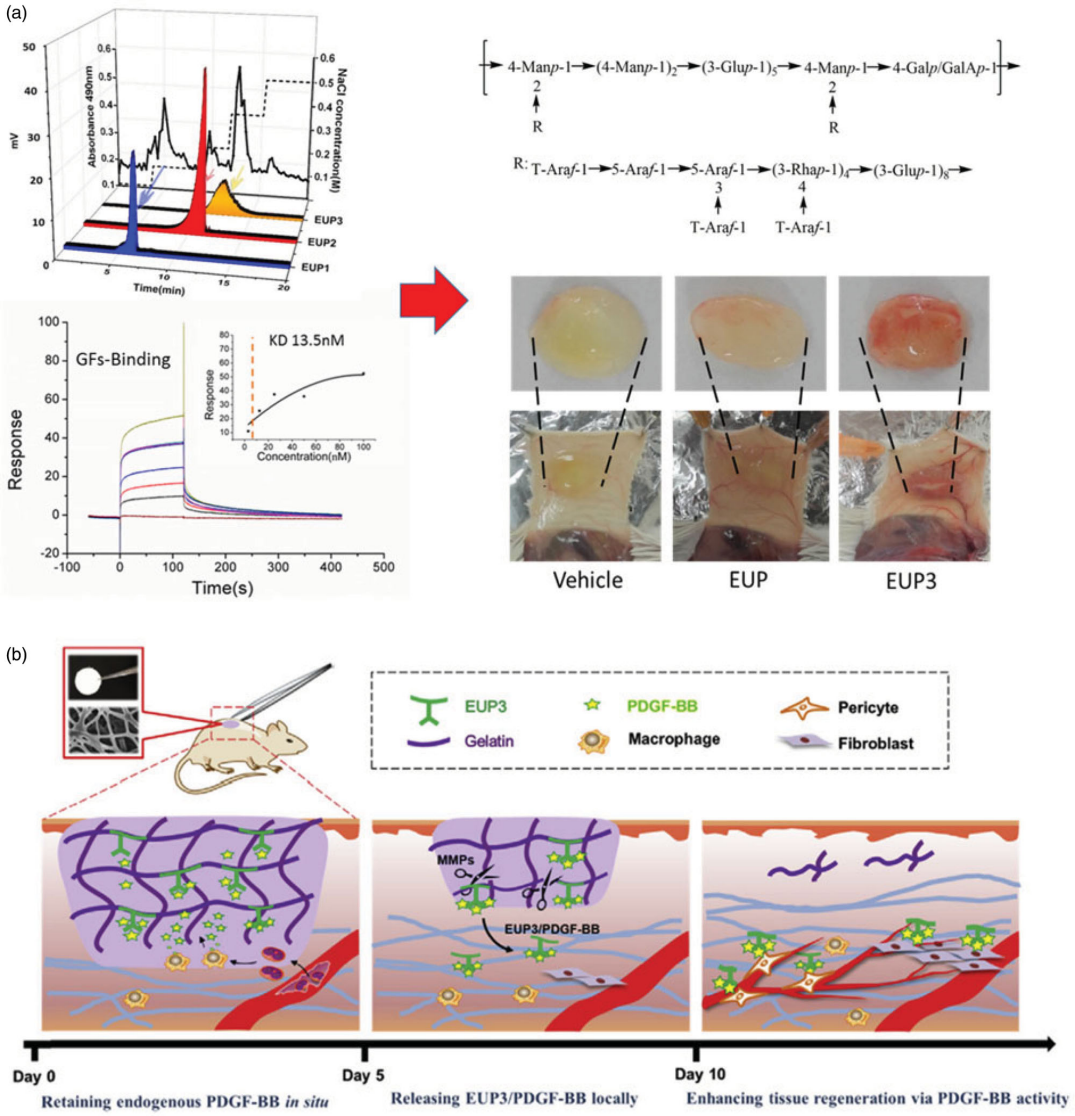
(a) a polysaccharide (EUP3) derived from *Eucommia ulmoides* was characterized, and showed potent capacity for binding with PDGF-BB. The *in vivo* experiment indicated that EUP3 could significantly promote vessel formation via sequestering PDGF-BB, compared with other groups. (b) Schematic illustration of the mechanisms of the designed ECM-mimetic sponge (EGS) for wound healing (Li et al., 2017).

In terms of controlling growth factors, new discoveries have been found and developed. For instance, Feng et al. obtained a novel polysaccharide form Konjac glucomannan, possessing affinitive for macrophages, and this polysaccharide could stimulate macrophages to release growth factors. This polysaccharide was further modified with heparin, and then they fabricated an injectable hydrogel scaffold consisted of KGM polysaccharide and heparin. They tested the efficacy of this scaffold in promoting angiogenesis. The results indicated that this scaffold based on polysaccharides had a potent prospect in tissue engineering (Feng et al., 2017). Additionally, except for KGM polysaccharide, Li et al. obtained a series of functional polysaccharides from herbs, expecting that they could have outstanding properties of controlling growth factors. They purified a polysaccharide from *Eucommia ulmoides*, named EUP3, containing a certain percentage of galacturonic acid. Unlike polysaccharides from animals such as glycosaminoglycans, EUP3 polysaccharide had no significant affinity for VEGF and bFGF, but had a clear binding affinity for PDGF-BB. Furthermore, Li et al. changed EUP3 polysaccharide into a growth factor-affinitive scaffold using electrospinning technology. The results demonstrated that this engineered scaffold based on EUP3 polysaccharide could enhance angiogenesis and promote wound healing via binding PDGF-BB (Li et al., 2016, 2017). As we mentioned above, polysaccharides from the Chinese medicinal herbs are special and have a promising potential for the applications in tissue engineering. Compared with other herbal polysaccharides, they have better mechanical properties and biological activities. Moreover, the polysaccharides avoid the risk of immune response and other side effects. Therefore, these polysaccharides exhibit a promising prospect, which have specific properties, can be used for growth factor delivery in tissue engineering (Figure 6).

## 3. New biodegradable polymers in tissue engineering

**Figure 7.**
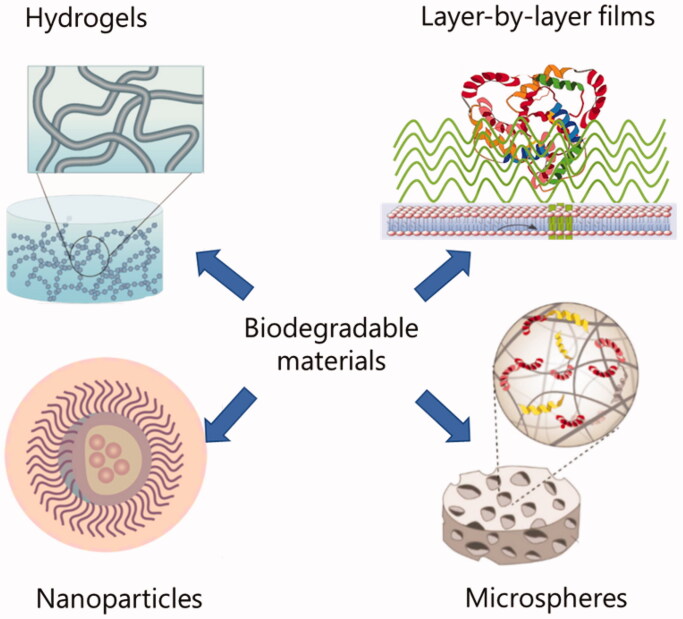
Nanoparticles (Saito & Takaoka, 2003), Scaffolds, Microspheres (Dinoro et al., 2019), Hydrogels (Hachim et al., 2019) based on biodegradable materials for delivering growth factors.

Many new biodegradable polymers were expected to use for drug delivery, which were found to consistently induce ectopic bone formation when they combined with GFs and were implanted into the muscles of experimental animals (Saito & Takaoka, 2003). Given the challenge in growth factor delivery, the potential to accurately control the delivery process of growth factors is prospective. Nevertheless, it is a very challenging problem because multiple problems must be solved in order to develop the fine system (Sun et al., 2019). A vital requirement for a tissue engineering scaffold is that it degrades and resorbs at a suitable rate. New degradable polymers have many advantages as scaffold materials in tissue engineering. Among the advantages of polymers, the ability to tailor mechanical properties and degradation kinetics is very useful. The polymers are also attractive because they can be changed into various delivery systems with the required morphologic features. Furthermore, the polymers can also be modified with chemical functional groups which can induce tissue regeneration. There are many biodegradable synthetic polymers such as poly (glycolic acid), poly (p-dioxanone), and so on, which have been widely used in clinic. This chapter describes the effects of nanoparticles, hydrogels, and layer-by-layer film assembly systems based on biodegradable polymers on growth factors delivery (Figure 7).

### 3.1. Nanoparticles for controlling growth factors

Nanotechnology has had a significant impact on a variety of therapeutics for decades. Advances in materials and formulation have produced safer and more efficient delivery of a myriad of drugs (Barclay et al., 2019). Targeted delivery strategies ensure a specific action and reduced systemic side effects. The flexibility and specificity of nanoparticle-based delivery systems provide an opportunity for targeting the immune system to initiate potent immune response.

The neuron growth factor (NGF) has the effect of restoring nerve damage and can regenerate the nerve. However, the effective delivery of drugs through the blood-brain barrier is still a major challenge. Lu et al. have invented an effective delivery system that enables NGF delivery to pass through the blood-brain barrier smoothly. The study selected a novel nanocarrier to encapsulate nerve growth factors and release in the nervous center system. In detail, this nanocapsule selected 2-methacryl phosphamidoline (MPC) as a monomer and polylactic acid (PLA) diacrylate as a cross-linking agent, which gathered around the NGF molecules through electrostatic interaction, and further transferred NGFs into a thin layer, which enclosed NGFs into the capsule. Due to the similarity of MPC with choline and acetylcholine, the capsule can be intravenously administered and recognized by choline transport protein and niacin acetylcholine receptor (nAChRs). Then the encapsulated neurotrophic factors can be released through the blood-brain barrier. This delivery system can effectively prolong the half-life in blood circulation without causing systemic reactions, and continuously release NGFs in damaged areas of the nervous system and reduce the occurrence of adverse reactions. Rajam et al. (2011) conducted a novel strategy, which prepared chitosan nanoparticles (CNP) and combined them with growth factors including epidermal growth factor (EGF) and fibroblast growth factor (FGF). The experimental results indicated that chitosan nanoparticles can be used as a dual growth factor delivery system in tissue engineering.

### 3.2. Hydrogels and microspheres for controlling growth factors

Hydrogel and microspheres are vital platforms for growth factor delivery, which usually have mechanical properties like those of many tissues, so they do not cause a strong inflammatory response through mechanical stimulation. In addition, the property of high-water content provides a means of releasing large growth factor molecules by diffusion in the water region between the polymer chains. The structure and mechanical properties of hydrogels can be regulated by a variety of different chemical methods. They can be made into injectable forms, such as nanoparticles, thixotropic systems or *in-situ* chemical or physical cross-linked polymers, so they are easily implanted into tissues through minimally invasive means (Amsden, 2015). It has been discovered that growth factors released from various hydrogel formulations have been designed, which binding growth factors with the hydrogel polymer network. Kobayashi et al. (2017) indicated that hydrogels could be explored for targeting delivery and controlled release of bFGF. The experiment results demonstrated that hydrogels have a better effect than solution injection alone. Rensburg et al. reported that hydrogels covalently modified by heparin could continue to deliver active growth factors, which also acted as a matrix significantly increasing angiogenesis *in vivo* (van Rensburg et al., 2017). Similarly, Jason et al. showed that degradable PEG hydrogel significantly influenced osteoblast differentiation by transmitting growth factors for osteoinduction. The results of all the study have demonstrated the potential of hydrogels to deliver growth factors, providing a reference for growth factor delivery strategies with endless potential (Rajam et al., 2011). Perez et al. (2014) developed novel microcarriers made of sol–gel-derived bioactive glasses for delivering growth factors for cultivating stem cells in bone tissue engineering. Basic fibroblast growth factor (bFGF) was incorporated into the microcarriers and showed a sustained release pattern, which promoted cell adhesion and proliferation on the bFGF-loaded microcarriers. It is indicated that the engineered mesoporous bioactive glass microspheres represent a new class of growth factor delivery carrier, potentially useful in tissue engineering.

### 3.3. Layer-by-layer films for controlling growth factors

In general, biomedical delivery systems require good mechanical functions to locally release growth factors to promote tissue response, and the perfect systems are deemed to achieve these aspects simultaneously with a single material. One way to fulfill it is to select a material with appropriate mechanical properties and then use a layer-by-layer assembly method to coat the surface with a film containing growth factors. The laminar assembly of the film on different substrate surfaces is based on alternating electrostatic adsorption of polyelectrolytes with opposite charges in aqueous solution (Amsden, 2015). Since growth factors have a net charge in an aqueous solution, they are easily incorporated into the film. The incorporation of the membranes depends on the isoelectric point of the growth factor and the pKa value of the polyelectrolyte. The release of growth factors from these components is a complex process that is influenced by the strength of the growth factors bound to the polyelectrolyte, the diffusivity of growth factors in the polyelectrolyte layer, and the degradation rate of the polyelectrolyte. This method has many advantages including basing on the water and no use of potentially toxic organic solvent in the assembly of the film. Certainly, this approach also has disadvantages. To achieve continuous release and minimal fracture effect, a large number of layers are usually required, which requires a lengthy preparation process. Kaminski et. al studied polymer-coated liposomes to deliver growth factors. Three-dimensional layer-by-layer structures of xanthan gum and galactans on liposome templates of dimethyl bromide (DODAB) have been studied and characterized. The experimental results showed that double-coated nanoparticles increase the continuous release of epithelial growth (EGF) by up to five times (Kaminski et al., 2016).

## 4. Conclusions

At present, precious growth factor delivery has exhibited a potential prospect in tissue engineering. With the tremendous development of novel biomaterials, it will increase our capacity to recapitulate tissue repair and regeneration through the precious delivery of various growth factors. Among these, the most commonly used biomaterials are natural polysaccharides and its analogues, which can be fabricated into scaffolds through versatile technologies. Polysaccharides derived from animals and herbs are natural biomaterials which are easily obtained. Among theses, a series of polysaccharides have been explored into functional biomaterial scaffolds for delivering growth factors. However, although a lot of polysaccharides were acquired and used as various biomaterial scaffolds in tissue engineering, the mechanism of polysaccharides interacting with bodies was still obscure due to the complicated structures. The detailed mechanisms and relationship between structure and activity should be observed further. Moreover, how the scaffolds made by polysaccharides exert functions is still a challenge. Acquisition of high purity and clear structure of polysaccharides is also difficult. Additionally, as for the application of functional polysaccharides in tissue engineering, although lots of researches have been performed for exploiting the functional scaffolds, there is still a long way to transform from research to clinic. In addition, there are still many limitations including the immunogenicity of scaffolds, low stability and high cost. In the future, we should develop the polysaccharide scaffolds with the requirements of clear structure, solid bioactivities, biocompatibility and security. Besides, the use of nanomaterials for delivering growth factors in tissue engineering is also extensively existing and multiple materials showed many advantages, where the materials can provide controlled and precise growth factors delivery. Although the same problem of transformation from research to clinic is existing, further development of scaffolds based on nanomaterials could be a worthy try. Meanwhile, many other fields could readily obtain a benefit from the utilization of biomaterials as carriers for delivering growth factors to the target tissues *in vitro* and *in vivo*.
